# Case Report: Innovative surgical management of ileal neobladder fistula after radical cystectomy

**DOI:** 10.3389/fonc.2025.1635458

**Published:** 2025-10-08

**Authors:** Zhiyang Ma, Xiaopin Ji, Wenbin Rui, Xiaojing Wang

**Affiliations:** ^1^ Department of Urology, Ruijin Hospital, Shanghai Jiao Tong University School of Medicine, Shanghai, China; ^2^ Department of General Surgery, Ruijin Hospital, Shanghai Jiao Tong University School of Medicine, Shanghai, China

**Keywords:** bladder cancer, cystectomy, fistula, preoperative diagnose, urinary diversion

## Abstract

An ileal neobladder fistula is a rare but serious complication of radical cystectomy with orthotopic neobladder reconstruction. Owing to its low incidence, the challenges posed by dense adhesions, and the risk to urinary function, there is little consensus on optimal management. A 68-year-old male with low-grade non-muscle-invasive bladder cancer underwent transurethral resection, followed by radical cystectomy and total laparoscopic orthotopic neobladder reconstruction. One month after surgery, the patient developed fecaluria. Imaging revealed a fistula between the neobladder and the ileum. Proximal ileostomy was performed to eliminate fecaluria. Seven months later, an open surgical repair was performed. Severe adhesions around the original ileal anastomosis and neobladder apex precluded safe separation. Rather than risking neobladder injury and loss of capacity, the surgical team preserved approximately 2 cm of the adherent ileum and restored intestinal continuity using overlap anastomosis. Postoperative recovery was uneventful, and follow-up revealed no recurrence of the fistula. This case demonstrates that the rapid identification and staged management of ileal neobladder fistulas can ensure favorable outcomes. The innovative surgical approach described here is effective in preserving both neobladder function and intestinal integrity and provides a viable, less invasive alternative for patients presenting with complex adhesions.

## 1 Introduction

Radical cystectomy (RC) is the standard treatment for patients with muscle-invasive bladder cancer (MIBC) and high-risk non-muscle-invasive bladder cancer (NMIBC) (1). Among the available post-RC reconstructive techniques, orthotopic neobladder (ONB) reconstruction has gained significant popularity among appropriately selected patients. ONB allows urinary continence and voiding through the urethra, thereby improving the quality of life compared to other forms of urinary diversion, such as ileal conduits or continent reservoirs (2–4). ONB is a technically demanding procedure associated with unique complications and challenges that require careful consideration. The development of ileal neobladder fistula (INF) is an uncommon but serious complication, with an approximate incidence of 0.7% (5).

Reports on the diagnosis and treatment of INF-related conditions are limited and inconsistent. In this manuscript, we reported a case of INF after ONB which was definitively diagnosed and successfully treated using a novel surgical technique.

## 2 Case presentation

A 68-year-old male, underwent transurethral resection of a bladder tumor (TURBT) at our hospital. During TURBT, the tumor exhibited diffuse development throughout the bladder with a broad base. Lesions with extensive cauliflower-like growth were observed around both ureteral orifices of the trigone. The tumor diameter was over 3 cm. Complete resection is considered unsafe because of the high risk of bleeding, ureteral orifice injury, and residual disease. Therefore, the lesions were classified as endoscopically unresectable. Only diagnostic resection was performed. Pathological results indicated low-grade, non-invasive urothelial carcinoma. In summary, this patient belonged to the high risk group. According to EAU guidelines, for this group of patients, especially when lesions threaten the ureteral orifices or when complete TURBT cannot be achieved to permit adequate staging and risk stratification, initial RC is considered to be a strong option to optimize oncologic outcomes. We discussed with the patient the management he could choose and the associated advantages and complications. Staged TURBT and initial RC could be considered. RC was chosen considering the possibility that staged TURBT might still fail to achieve complete resection and high surgical risk. Urinary diversion options after cystectomy were reviewed, including ileal conduit, continent cutaneous diversion and ONB. The patient had no prior history of pelvic radiotherapy and no significant systemic comorbidities like diabetes. Renal function was within normal limits. The patient was willing to void in a natural way and did not have signs of urethral stricture. No tumor lesions near the bladder neck or prostatic urethra met the indications for ONB. Therefore, after thorough communication with the patient, we decided to use a segment of the ileum as an ONB and an endoscopic linear-cutting stapler to restore ileal continuity. A few weeks later, on 11 February 2022 we performed total laparoscopic RC combined with U-shaped ONB. The surgery was successful and the postoperative pathology confirmed a negative urethral margin.

**Figure 1:**
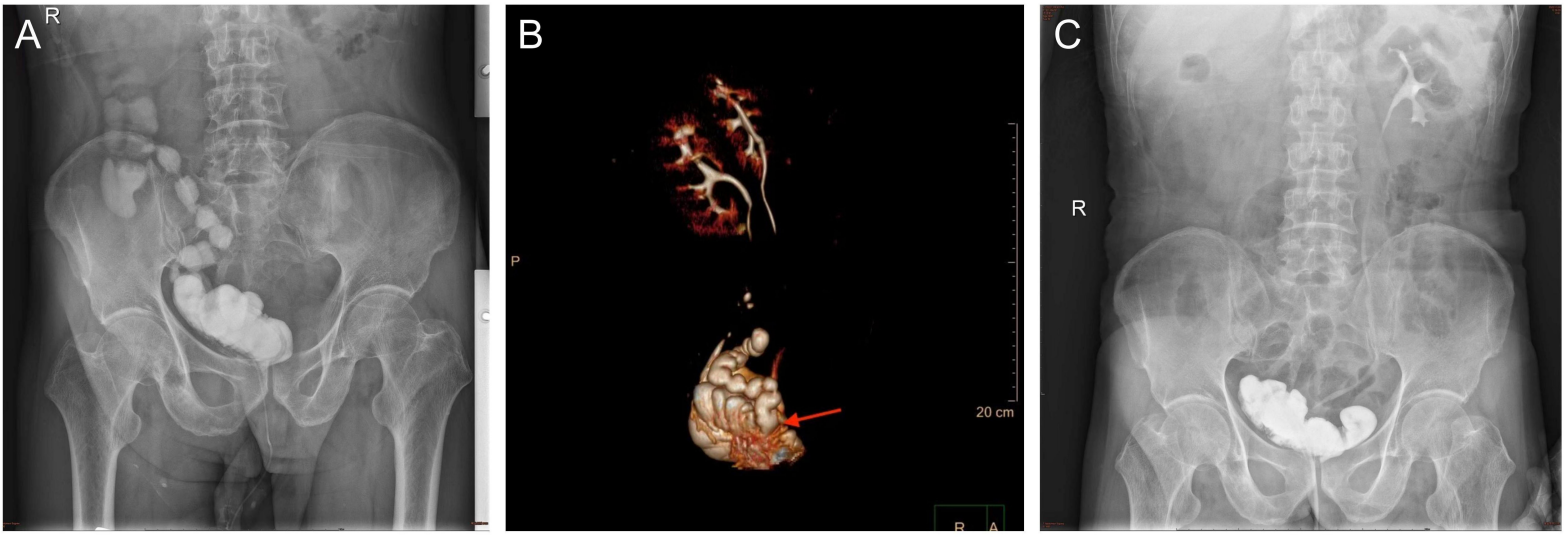
**(A)** The image of cystography examination showed the contrast agent was found in the ileum and right colon before the surgery. **(B)** Computed tomography urography reconstructed the spatial position of the neobladder and ileum. **(C)** The image of cystography examination showed the contrast agent was not found in the ileum and right colon after the surgery.

One month after surgery, the patient returned to the outpatient clinic because of the presence of fecal material in his urine. Cystography revealed that the contrast agent was present in the bladder, ileum, and right colon. Computed tomography urography (CTU) was performed, which explicitly lead to the suspicion of INF associated with ONB (**Figures 1A, B**). In patients with frequent tract infections, we scheduled an elective fistula repair operation. First, we performed proximal ileostomy on 21 March 2022 to prevent continuous fecaluria and ensure an aseptic environment in the neobladder. Fecaluria did not occur immediately after ileostomy, further confirming our hypothesis of INF.

**Figure 2:**
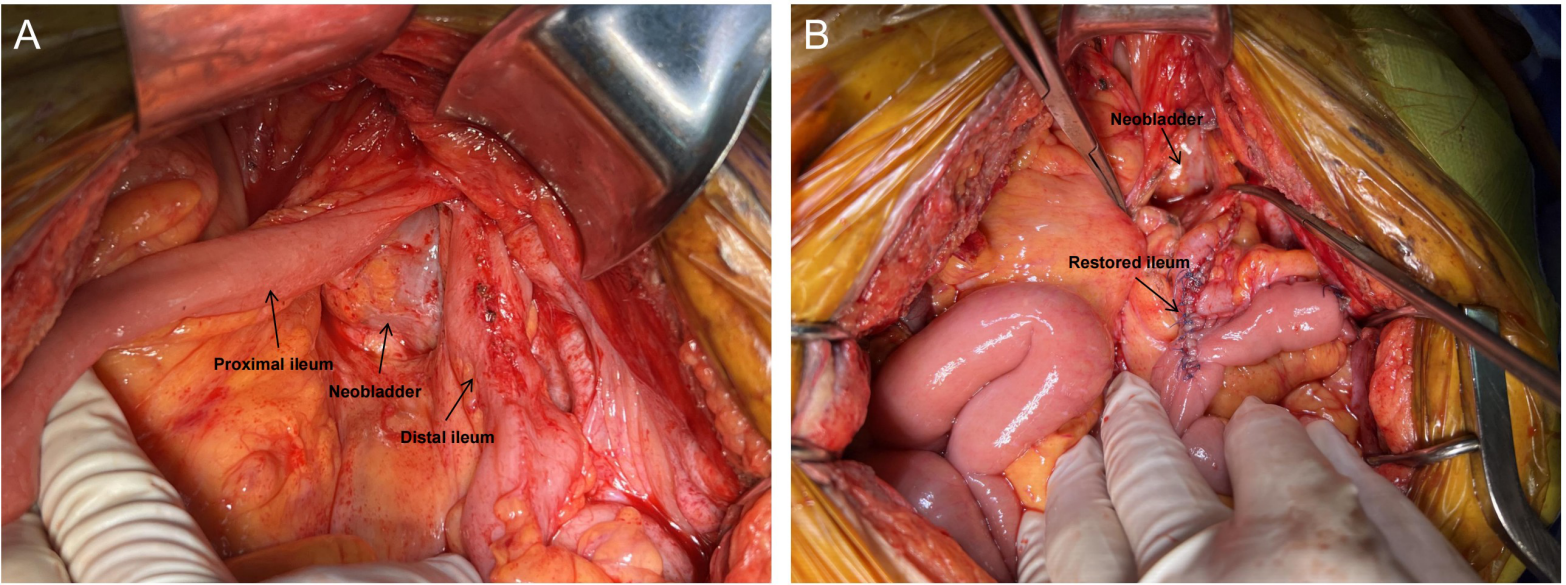
Intraoperative picture. **(A)** The original ileum anastomosis and the apex of the neobladder were adherent to the pelvic wall. **(B)** The adhered portion of the ileum was preserved and the original ileum was restored.

Seven months after the ileostomy, we decided to repair the fistula. During these months, there were no symptoms such as urinary infections or difficulties when urinating. On 20 October 2022 we performed laparotomy and found that the original ileum anastomosis and apex of the neobladder were adherent to the pelvic wall (**Figure 2A**). The adhesions were severe and difficult to separate. We believe that forced separation may have resulted in the removal of a significant portion of the neobladder tissue, affecting its postoperative capacity. In addition, forced separation can easily damage the ureteral orifices of the neobladder, necessitating subsequent re-implantation of the ureters into the neobladder. Therefore, we preserved the adhered portion of the ileum. We mobilized the proximal and distal ends of the original ileum anastomosis and transected them as close to the neobladder as possible, leaving an approximately 2 cm segment of the ileum adherent to the neobladder. Original ileal continuity was restored using an overlapping technique (**Figure 2B**). The residual ileum ends on both sides were sutured and reinforced, and blood supply was ensured. The previously created proximal ileostomy was closed. No abnormalities were observed during the neobladder water injection test. Methylene blue was administered via an intravenous drip, and the urine collected in the drainage bag appeared pale blue with no pale blue color changes observed in the surgical field, indicating good closure of the postoperative neobladder.

**Figure 3:**
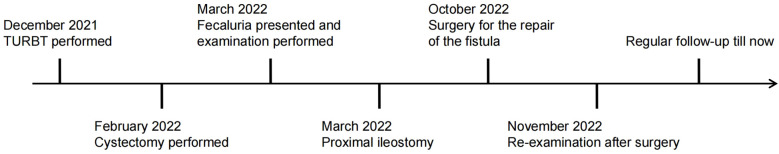
Timeline with relevant data from the episode of care.

Postoperative recovery was good, and the patient was discharged seven days after the surgery. Twenty days later, cystography revealed that the contrast agent was not present outside the neobladder (**Figure 1C**). The patient has been regularly followed-up. The patient did not experience fecaluria or digestive discomfort. The daytime and nighttime urinary continence rates were 100% and 75%, respectively. A timeline illustrating the information in this case is presented in **Figure 3**.

## 3 Discussion

ONB are associated with unique complications and challenges that require careful consideration. Neobladder-vaginal fistula and INF are potential complications of ONB. In large volume medical centers, the incidence rates of neobladder-vaginal fistulas range from 3% to 6% (6). In another large-cohort clinical study, INF developed in 12 (2.2%) of 553 patients who underwent ONB (7). The formation of this type of abnormal communication between the neobladder and adjacent intestinal segments in INF is multifactorial. Surgical factors such as ischaemia, tension at anastomosis, or radiation-induced tissue damage, and patient-related factors such as infections, chronic inflammation, diabetes, or malnutrition impair wound healing and increase the risk of developing INF (8, 9). A history of radiation therapy is a predictor as well (10). Secondary complications such as urinary tract infections, bladder stones, and postoperative inflammation exacerbate this risk (11). Chronic irritation or urinary leakage can damage the adjoining tissues over time, breaking down the mucosal barriers and precipitating fistula formation. Our patient did not have diabetes or bladder stones. He had no prior history of pelvic radiotherapy. Based on this discovery during surgery, we suspected that the combination of a stapled side-to-side ileal anastomosis, mesenteric mobilization, and postoperative pelvic descent due to gravity likely facilitated direct staple-line adhesion to the neobladder apex. Adhesions render local tissue more susceptible to infection, ischaemia, and mechanical stress, gradually leading to the development of a fistula over time.

INF often presents atypically with symptoms such as fecaluria, recurrent urinary tract infections, watery stools, and abdominal pain, which may lead to misdiagnosis and disease progression. Accurate diagnosis requires advanced imaging techniques, such as cystography and enterography (12). The patient immediately underwent cystography examination after noticing fecal materials in the urine without other discomfort and underwent proximal ileostomy before secondary complications emerged. We conclude that the rapid identification of INF contributes to favorable patient prognosis.

The management of INF traditionally involves either conservative measures or surgical intervention, depending on the severity of the symptoms and the extent of the anatomical defect. Conservative management includes catheter drainage, temporary ileostomy, and antibiotic therapy to mitigate inflammation and infection (13). Also, a low-residue diet such as corn or whole spices was easy to employ and the patient demonstrated excellent compliance. It was suitable for patients with good nutrition status and without signs of local or systemic infection, thus avoiding temporary ileostomy^i^. However, this approach rarely resolves fistulas definitively. Spontaneous healing probability was <10% because these patients often develop complications such as malnutrition, immunosuppression and sepsis due to the formation of INF, leading to impaired fistula healing (13–15). Also, conservative management can be used as a complementary strategy for surgical repair to help patients achieve optimal condition for surgery and postoperative recovery (16).

Surgical intervention, the mainstay of definitive treatment, traditionally requires excision of the fistulous tract, ileal resection, and re-anastomosis. Techniques such as partial resection of the neobladder or meticulous separation of adherent tissues have demonstrated varying levels of success (5). Partial neobladder excision can be performed in adhesion-dense fields. On the bladder side, excision of the fistula with a two-layer closure and transurethral catheter drainage for 10–14 days is sufficient. On the intestinal side, depending on the extent of the fistula, either excision with a two-layer closure or a small-bowel segmental resection may be necessary. If the ureter orifices are at risk, ureteral reimplantation may be required. The ureter-enteric anastomosis could be wrapped with a pedicled omentum majus flap for protection (17). Forced dissection can be dangerous, increasing intraoperative bleeding and infection risks, reducing neobladder capacity, and leading to postoperative ureteral anastomotic stricture. A laparoscopic or minimally invasive approach to fistula repair offers the advantages of reduced morbidity and shorter recovery times. However, it is limited by the extent of adhesion and tissue damage, which may render laparoscopic dissection unsafe (18). Other surgical options include neobladder takedown and conversion to external diversion. The operation can be simplified, but the patient will lose the ability to void in a natural way, and their quality of life will significantly worsen. The comparison among the traditional management of INF was shown in **Table 1**.

**Table 1:** The conventional management strategies and their key steps, indications, relative advantages and limitations.

Strategy	Key steps	Indications	Advantages	Limitations
Conservative management	1. Urinary drainage; 2. Temporary proximal ileostomy; 3. Broad-spectrum antibiotics; 4. Comorbidities control; 5. Imaging surveillance;	1. Small, low-output fistula without sepsis or severe contamination; 2. Early postoperative fistulas; 3. A staged approach to stabilize infection;	1. Minimal invasion; 2. Sepsis control; 3. Local inflammation downstaging;	1. Low definitive closure rate; 2. Insufficient as sole therapy; 3. Risk of persistent urinary infections and malnutrition; 4. Possibility of transition to surgery;
Standard surgical repair	1. Identification of fistulous tract; 2. Segmental ileal resection of involved bowel and re-anastomosis; 3. Partial neobladder resection; 4. Re-implantation of ureters if orifices are at risk; 5. Integrity testing of neobladder closure;	1. Persistent fistula after conservative measures; 2. High-output fistula, recurrent infections or stones; 3. Clear fistulous communication; 4. Adequate patient status for surgery;	1. Definitive therapy with high likelihood of closure; 2. Directly addressing adherent tissue;	1. Technically challenging in dense adhesions; 2. Risk of bleeding and organ injury; 3. Potential reduction in neobladder capacity; 4. Risk to ureteral orifices;
Conversion to external diversion	1. Take down neobladder; 2. Construct alternative incontinent diversion; 3. Ureteral re-implantation into conduit;	1. Refractory fistula despite repair; 2. Small-capacity neobladder; 3. High surgical risk of preserving the neobladder; 4. Patient preference prioritizing simplicity;	1. Simplifying anatomy and reducing recurrence risk; 2. Effective infection control when neobladder is unsalvageable;	1. Loss of natural voiding; Impact on quality of life; 2. Psychological and lifestyle adjustments;

The technique proposed in this report is a scenario-specific, function-preserving alternative to established surgical principles. By preserving the ileal adherence to the fistula and anastomosing the ileum end-to-end directly, there was no inadvertent injury to the neobladder, ureters or surrounding organs. This not only reduces perioperative risk and surgical complexity but also preserves the functional capacity of the neobladder, which is critical for maintaining the quality of life. Moreover, this technique eliminates the need for extensive ileal resections. As mentioned in another case, the surgeon established a bypass 10 cm from the fistula, and the patient in Case 1 experienced fecal recurrence and underwent another surgery (19). We left only a 2 cm segment of the ileum to minimize the functional compromise of ileal absorption and motility and to reduce mucus entry into the neobladder, which may otherwise increase the infection risk. Additionally, we performed an initial proximal ileostomy to provide a more favorable environment for INF repair, thereby lowering the risk of inflammation and recurrence. This technique is reproducible under similar conditions. The indications include absence of uncontrolled sepsis, optimization after proximal fecal diversion and dense adhesions at the neobladder apex and patient preference for neobladder retention. Procedural elements, such as short adherent segment preservation, overlap anastomosis and intraoperative integrity testing, are standard and widely applicable in experienced hands. However, careful patient selection and the surgeon’s judgment are crucial. The contraindications include suspected neobladder ischemia or extensive wall involvement necessitating resection, inability to mobilize healthy ileum for a tension-free anastomosis and recurrent fistula where takedown and conversion may be more appropriate. These details specify when and how the technique can be reproduced and what outcomes can be reasonably expected under comparable anatomical and clinical conditions.

Most importantly, preventing the development of INF is the key to all intervention measures. During surgery, ensuring adequate mesenteric length and orienting the ileal anastomosis away from the neobladder apex can prevent direct contact between high-risk fistula sections. Burying a linear stapler within the serosal layer is also helpful. Prior to abdominal closure, inspection for potential staple exposure near the neobladder is necessary.

Because INF is a low-incidence postoperative complication, we report only one patient who underwent this technique. Although the technical steps are standard and reproducible in similar scenarios, validation in larger cohorts or multi-institutional series is required to compare the outcomes with those of conventional strategies. The observation period of this study was relatively short. Longer surveillance periods are required to assess late complications and functional stability. Although continence outcomes were favorable, future studies should incorporate urodynamics to objectively assess reservoir function and pressure.

## 4 Conclusion

This case demonstrates that an innovative surgical approach that preserves the adhered ileal segment can effectively manage ileal neobladder fistulas, minimize complications, and maintain neobladder capacity, offering a valuable treatment alternative for similar complex postoperative cases.

## Data Availability

The original contributions presented in the study are included in the article/supplementary material. Further inquiries can be directed to the corresponding authors.
